# Combined Schwannoma and Kappa-Restricted Plasma Cell Neoplasm: A Case Report and Review of the Literature

**DOI:** 10.1155/2021/8825316

**Published:** 2021-01-15

**Authors:** Jeffrey John Cannatella, Soumya Pandey

**Affiliations:** ^1^Department of Pathology, University of Nebraska Medical Center, 983135 Nebraska Medical Center, Omaha, NE 68106-3135, USA; ^2^Department of Pathology, University of Arkansas for Medical Sciences, 4301 West Markham Street, Mail Slot 502, Little Rock, AR 72205, USA

## Abstract

The patient is a 78-year-old woman with a popliteal soft tissue mass that was tender to palpation with shooting pain on physical examination. A schwannoma was seen on biopsy with subsequent excision demonstrating a concomitant kappa-restricted plasma cell neoplasm. Follow-up did not show evidence of a systemic plasma cell neoplasm. MRI studies showed no evidence of focal lesions, although PET-CT revealed presence of multiple lytic lesions. The patient is currently being monitored every six months. This case is the first kappa-restricted plasma cell neoplasm reported in association with a schwannoma and the first reported in the extremities.

## 1. Introduction

Schwannomas are relatively common peripheral nerve sheath neoplasms that comprise approximately 5% of benign soft tissue tumors in some centers [1]. Schwannomas predominantly consist of Schwann cells, which originate from the neural crest and lack Merlin protein expression [2–4].

In contrast, plasma cell neoplasms are derived from terminally differentiated B cells that are monoclonal, a feature often demonstrated by light chain restriction [5]. Extraosseous plasmacytomas (EP) comprise approximately 4% of all plasma cell neoplasms with the majority occurring in the upper aerodigestive tract [6]. EPs and plasma cell myelomas have similar genetic abnormalities, and progression to plasma cell myeloma occurs in approximately 15% of cases [5].

Despite their seemingly disparate origins, there are two case reports of patients with both schwannoma and monoclonal plasma cell populations [7, 8]. Damasena et al. described a marginally resected retroperitoneal mass in a 54-year-old man diagnosed as a schwannoma with an intermixed lambda-restricted plasma cell population [7]. Serum protein electrophoresis (SPEP), skeletal survey, and bone marrow biopsy were unremarkable, and no evidence of myeloma was noted 12 months after surgery [7].

Plaut et al. reported a cerebellopontine angle mass in a 45-year-old woman [8]. Microscopic evaluation of the subtotal excision demonstrated a schwannoma with an intermixed lambda-restricted plasma cell population. Imaging and SPEP were unremarkable. Bone marrow biopsy was not performed due to patient comorbidities.

Combined schwannoma and plasma cell neoplasms have rarely been reported in the literature. Herein, we present the first such case reported in the extremities as well as the first case with kappa-restricted plasma cells.

## 2. Case History/Examination

The patient is a 78-year-old woman with a right popliteal soft tissue mass initially noticed 6 months ago. Physical examination elicited shooting pain when the lesion was tapped. Pertinent laboratory results included mild normocytic anemia (hemoglobin 10.8 g/dL) and normal serum calcium (8.6 mg/dL). MRI study revealed a 6.5 cm soft tissue mass posterior to the distal femoral shaft. She underwent needle core biopsy of the mass, and microscopic examination was consistent with a schwannoma. She reported difficulty sleeping after the biopsy due to pain, and excision was performed. Intraoperatively, the mass appeared to involve a branch of the common peroneal nerve.

**Figure 1:**
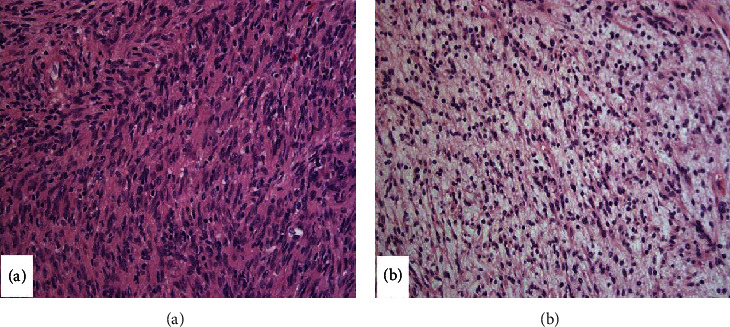
Legend: H&E section (20x) shows a spindle cell tumor composed of (a) hypercellular areas with vague nuclear palisading and (b) hypocellular areas with a myxoid background.

**Figure 2:**
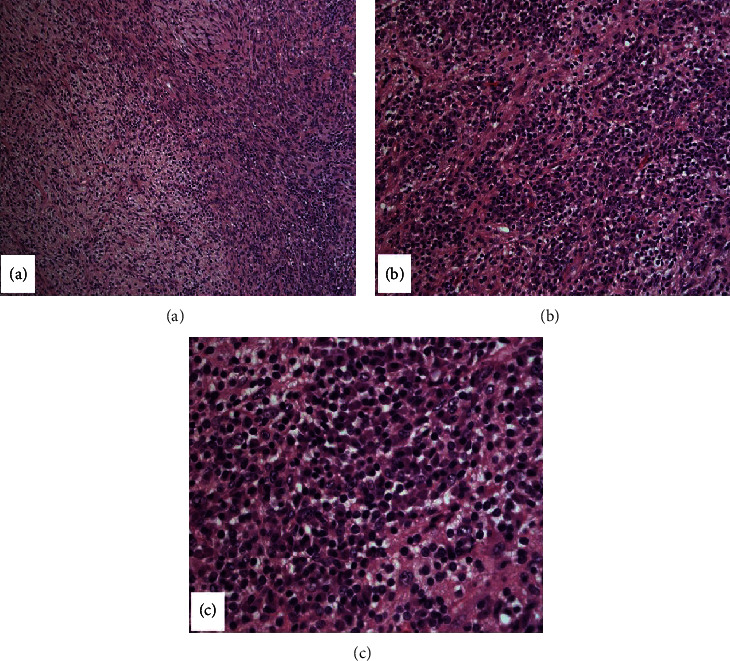
Legend:(a) H&E section (10x) showing schwannoma with palisading nuclei on the left and a plasma cell infiltrate on the right. Higher power view of the plasma cell infiltrate (b: 20x) (c: 40x) shows distinct clusters of small, mature-appearing plasma cells.

**Figure 3:**
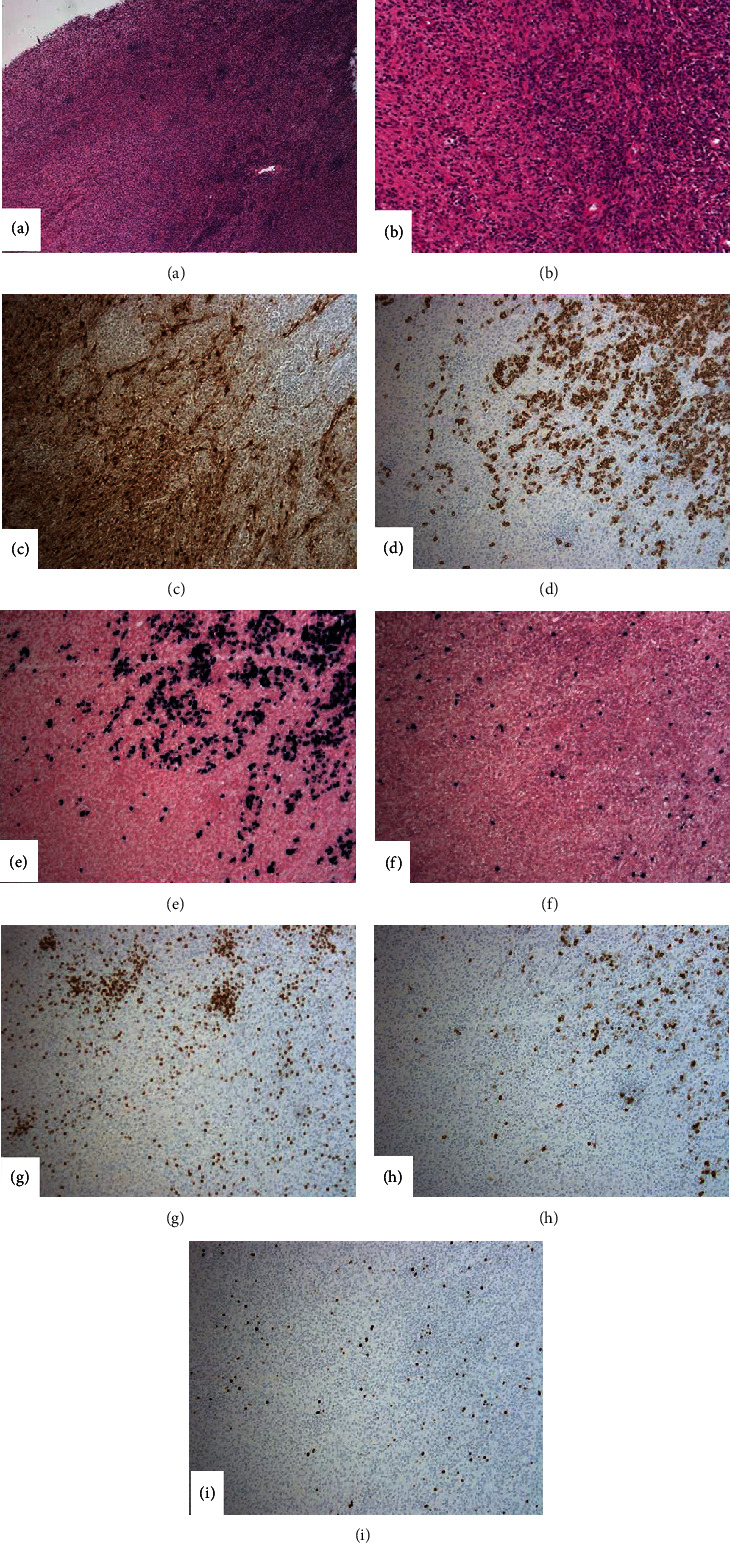
Legend: H&E section (a: 4X) and (b: 10X) showing schwannoma, with interspersed small lymphoid aggregates and a plasma cell infiltrate on the right. (c) S-100 showing diffuse and strong, cytoplasmic, and nuclear positivity in the schwannoma component on the left (10x). (d) CD138 immunostain (10x) showing plasma cells infiltrating into the schwannoma. In situ hybridization studies for kappa (e) and lambda (f) light chains (10x) show that the plasma cells are kappa-restricted. (g) CD3 (10x) highlights T cells scattered interstitially and within the lymphoid aggregates. (h) CD20 (10X) highlights few scattered B cells. (i) Ki-67 (10x) shows very low proliferative activity.

The gross specimen consisted of a lobulated soft tissue measuring 7.4 × 4.5 × 4.5 cm. Sectioning revealed heterogeneous tan-yellow and gelatinous cut surfaces. There were no overt arears of necrosis. Microscopic examination was again consistent with presence of schwannoma.  Figure 1 demonstrates a biphasic spindle cell tumor with alternating hypercellular (Figure 1(a)) and hypocellular areas (Figure 1(b)). The hypercellular areas were composed of spindled cells arranged in compact fascicles and showed vague nuclear palisading. The spindled cells within the hypocellular areas were more haphazardly arranged and showed a myxoid background. The spindled cells were narrow, elongated, wavy, and had inconspicuous nucleoli. Mitotic figures were not prominent, and there was no evidence of necrosis. Scattered small mature appearing lymphocytes and small lymphoid aggregates were noted. Additionally, prominent clusters of plasma cells were noted intermixed with the elements of the schwannoma throughout the lesion (Figure 2). The plasma cells were small and mature-appearing. A panel of stains were performed as depicted in Figure 3. S-100 showed diffuse and strong cytoplasmic and nuclear positivity in the spindle cells. The spindle cells were negative for CD34, SMA, desmin, and pancytokeratin (not shown). CD138 highlighted the plasma cell component, while kappa and lambda light chain in situ hybridization studies demonstrated kappa restriction. CD3 and CD20 highlighted scattered T and B cells, respectively, and distributed interstitially and within the small aggregates. Ki-67 showed low proliferative activity in both components.

## 3. Differential Diagnosis, Investigations, and Treatment

The biphasic nature of this spindled cell tumor, along with strong and diffuse S-100 staining, was consistent with patient's known diagnosis of schwannoma. The morphologic appearance (lack of mitosis and necrosis) and immunohistochemical staining pattern (lack of positivity for CD34, SMA, desmin, and pancytokeratin) made other spindle cell tumors (leiomyoma/leiomyosarcoma, malignant peripheral nerve sheath tumor, neurofibroma, pleomorphic hyalinizing angiectatic tumor) less likely. The differential diagnosis for the plasma cell lesion included a solitary extraosseous plasmacytoma, plasma cell myeloma, or marginal zone lymphoma associated with a schwannoma. To diagnose an extraosseous plasmacytoma, the lesion should have a clonal plasma cell population in a patient with no clonal plasma cells on bone marrow biopsy, unremarkable skeletal survey and MRI or CT, and absence of end-organ damage (i.e., CRAB symptoms) that are attributable to the plasma cell neoplasm [5].

## 4. Outcome and Follow-Up

A follow-up bone marrow biopsy did not identify a monoclonal plasma cell neoplasm on the microscopic evaluation or flow cytometry. Focal lesions were not seen by MRI; however, PET-CT described lytic lesions in the right ischium, right tibia, and numerous small lytic lesions in the calvarium. Over a 3-month period, pertinent laboratory results included stable normocytic anemia (10.8 g/dL), mildly increased serum creatinine (1.1-1.3 mg/dL), and normal serum calcium. Serum protein electrophoresis demonstrated a decrease in gamma region proteins (0.4 g/dL). Immunofixation did not show an IgA, IgG, and IgM paraprotein, and kappa and lambda free light chains did not show restriction (lambda/kappa ratio of 0.52). Urine protein electrophoresis and immunofixation were also negative for a monoclonal protein. Overall, these findings were not sufficient for a multiple myeloma diagnosis; however, the presence of lesions by PET-CT, even without confirmation of the lesions by MRI, prompted the follow-up evaluation in 6 months with repeat peripheral blood tests and PET-CT.

## 5. Discussion

Schwannomas and plasma cell neoplasms have disparate cells of origin. Despite this, two prior cases have been reported describing monoclonal plasma cell populations with schwannomas, and the literature also describes these neoplasms in association with other entities. For example, a case of schwannoma with a gastrointestinal stromal tumor (GIST) has been described [9]. Plasma cell neoplasms have been reported with renal cell carcinoma [10] and nasopharyngeal carcinoma [11]. Possible explanations for these collision tumors are largely speculative with three postulated general mechanisms—(1) coincidence, (2) tumors metastasizing into other tumors, and (3) both tumors arising from a common progenitor or from similar stimuli. The seemingly hospitable environment for metastatic deposits created by the slow growth rate and relatively rich vasculature in schwannomas lends some credence to mechanism two [7, 8]. Mechanism three offers alternative explanations, such as a plasma cell neoplasm arising from a reactive process to the schwannoma [7]. Cytokines, such as IL-6, could conceptually play a role. IL-6 production has been noted in acoustic neuromas [12], and IL-6 has been associated with renal cell carcinoma and plasmacytomas [10]. Further evaluation is needed to elucidate the true underlying mechanism.

Cellular schwannomas can have a subcapsular lymphocytic infiltrate. While a brisk subcapsular lymphocytic response was considered in this case, the large swaths of plasma cells prompted further evaluation with stains for kappa and lambda light chains and CD138. While a diagnosis of marginal zone lymphoma with prominent plasma cell differentiation was a consideration, the background lymphocytes were small without a notable population of monocytoid or centrocyte-like forms. However, the fresh tissue was not available for further evaluation with flow cytometry or FISH studies.

In conclusion, this is the third case reported in the literature of a plasma cell neoplasm associated with a schwannoma and the first case in an extremity and with kappa-restricted plasma cells. Similar to the prior cases, no systemic disease was found on the follow-up evaluation in our patient; however, the patient will continue be monitored every 6 months.
